# The strategic role of competency based medical education in health care reform: a case report from a small scale, resource limited, Caribbean setting

**DOI:** 10.1186/s13104-014-0963-1

**Published:** 2015-01-21

**Authors:** Jamiu O Busari, Ashley J Duits

**Affiliations:** Department of Pediatrics, Atrium Medical Center, Henri Dunantstraat 5, 6401 CX Heerlen, Netherlands; Department of Educational Development and Research, Faculty of Health, Medicine and Life Sciences, University of Maastricht, Maastricht, the Netherlands; Department of Medical Education, St. Elisabeth Hospital, Willemstad, Curaçao; Institute for Medical Education, University Medical Center Groningen, Groningen, the Netherlands; Red Cross Blood bank Foundation, Willemstad, Curaçao

**Keywords:** Competency, Resource-limited, Health care, Caribbean, Medical education

## Abstract

**Background:**

Curaçao is a Dutch Caribbean island with a relatively high aging population, a high prevalence of chronic diseases and a health care system that is driven by cost-containment. In 2009 the development of a new value-based health care (VBHC) system was initiated on the island, and a key role was identified for the St. Elisabeth Hospital as a (model) platform for implementing this initiative. We therefore decided to investigate for the requirements needed to build a health care environment that is conducive for change and capable of facilitating the smooth migration of existent services into an effective and sustainable VBHC system.

**Findings:**

Our findings revealed that our chosen approach was well accepted by the stakeholders. We discovered that in order to achieve a new value based health care system based on a reliable and well-organized system, the competencies of health care providers and the quality of the health care system needs to be assured. For this, extra focus needs to be given to improving service and manpower development both during and after formal training.

**Conclusions:**

In order to achieve a VBHC system in a resource-limited environment, the standard of physicians’ competencies and of the health care system need to be guaranteed. The quality of the educational process needs to be maintained and safeguarded within an integrated health care delivery system that offers support to all care delivery and teaching institutions within the community. Finally, collaborative efforts with international medical institutions are recommended.

## Background

In the last 10 years, the process of health care reform in several national health care programs has witnessed an increased need for complex and innovative approaches at the organizational, clinical and policy decision-making levels [1]. A closer look at many of these reform processes however, shows that little information is available on the specific role medical education plays (or can play) in aiding reform, and achieving high quality health care services [2]. There are reports in the literature that reflect a weak alignment between the roles of doctors and the health care needs in their communities. Also about how medical training programs are finding it difficult to deliver competent doctors, who are trained to effectively participate in modern patient and population-centered health care systems [3,4]. These developments apparently suggest that in order to achieve successful health care reform within a community, a favorable environment for change is necessary. Such an environment would need to be able to facilitate the development of relevant (and specific) competencies of health care professionals, needed to be able to function effectively within a modern health care system [1,4,5].

At present, several small scale and/or resource-limited communities are facing challenging times in achieving sustainable health care programs in their countries, due to limited financial resources. This is further complicated by the shortage of qualified health care professionals required to provide essential health care services within these communities. Extensive literature studies have shown that due to the high impact of local circumstances, no single strategy exists that can guarantee a successful approach and implementation of effective health care systems [6,7]. However, we are of the opinion, that contrary to the current views in the literature, implementation of a continuous learning organizational environment could serve as an important basis for effective health care reform in resource-limited settings. Especially when these are combined with clear descriptions of the organizational context for defining inherent and potential health care challenges within the community [4,8]. In order to illustrate this viewpoint, a description of a resource-limited environment currently facing challenges of health care reform is provided and how the incorporation of a structured educational framework formed an effective basis for implementing new health care strategies within the community.

### Context

Curaçao is a Dutch Caribbean island with an estimated population of 150.563 inhabitants [9]. It is characterized demographically by a relatively high aging population, a high prevalence of chronic diseases (e.g. diabetes, obesity, hypertension), and poor financial and human resources to enable the reform of its health care system that is currently being driven by cost-containment. Furthermore, the delivery of health care on the island is portrayed by a fragmented, unsynchronized and inefficiently functioning primary, secondary and tertiary health care system.

In terms of economic health, the average gross household income in Curacao in 2011 was 5.331 NAF (3013 USD) with a median yearly income of 3.500 guilders (1980 USD). The Modal household income was between 1.001 – 2.000 NAF (565–1130 USD) per month with about 17% of the household population belonging to this income category and another 13% below 1.000 NAF (565 USD) a month [10]. In 2012, the per capita GDP for the Netherlands Antilles was estimated by the United Nations as 18.360 USD with the per capita GDP of the World, USA and Western Europe estimated as 10.269, 51.163 USD and 43.313 USD respectively [11]. The Per capita GDP is a measure of the total output of a country and is a useful indicator for comparing the relative economic performance of one country to another, with a rise in per capita GDP signaling a growth in the economy and the country’s productivity. In 2006, the perinatal mortality rate in Curacao was slightly lower than the average within the Caribbean region (23.5 vs. 31/1000). However, this figure was notably higher than those found in countries in North America and Western Europe (7 and 13/1000 respectively) [12]. According to the WHO, the health status of a country can be evaluated based on the perinatal mortality rate, as this tends to reflect the standard of a country’s obstetric and pediatric health care [12]. Finally between 2012 and 2013, there was a decline in the infant mortality rate in Curacao from 11.3/1000 to 7.7/1000 live births [13].

With its historic lack of clear governmental policy regarding health care organization and planning, the assurance of optimal patient care and a sustainable health care system in Curacao, has been subjected to continuous threat. The associated increase in health care consumption, poor formal patient representation and increasing expenditure on the delivery of health services, have also been of little help in this area [14]. Based on the above points, the situation in Curacao fits the description of a country (island) with limited resources and a poorly developed health care system. Consequently, this has implications for the prioritization and distribution of available resources as well as, for the successful implementation of (new) educational and health care initiatives.

In 2009 the health care council of the island initiated a new health care movement, advocating the development and implementation of a new and value-based approach that would reform the island’s health care delivery system. As a result, it became imperative to identify appropriate strategies and essential local conditions that would be needed to achieve the successful implementation of a new value-based health care system [15]. The outcome of this investigative process, as evidenced by the official reports by the national health care council, identified a key role for the St. Elisabeth Hospital as a (model) platform for implementing the new health care reform initiative [16–18].

The St. Elisabeth Hospital (SEHOS) is the sole general hospital of Curaçao and with its 536 beds, is the largest health care institution within the Dutch Caribbean area. In addition to the SEHOS, there are 2 smaller surgical clinics (40 & 45 beds respectively), 1 psychiatric hospital (200 beds), a 146 bed rehabilitation hospital, 2 nursing homes and a maternity clinic that serve the inhabitants of the island [19]. As of December 2012, a total of 284 medical professionals were reported to be practicing on the island. One hundred and forty were registered as medical specialists, 110 general practitioners, 294 allied health care professionals (e.g. physiotherapists), 6 registered midwives and 54 dental professionals [20].

The SEHOS provides services in all major clinical specialties, and also offers adult, pediatric, and neonatal intensive care. The hospital, as an educational setting, is affiliated to a number of tertiary medical institutions in the Netherlands and provides accredited residency and pre-residency training for Dutch medical students of the University Medical Center Groningen (UMCG) [21,22].

Prior to the health council’s project in 2009, the new (G2010) curriculum of the UMCG was implemented in SEHOS as a novel competency-based training program for medical residents and students. A specific educational strategy was developed and used to implement the curriculum as well as evaluate and monitor its impact on the quality of the professional training [22]. As a result, in evaluating the suitability of the SEHOS as a model platform for the health care reform initiative, a systematic analysis of the hospital’s characteristics was performed using a medical education quality assurance project. This project focused on assuring the delivery of competency based training to health professionals in Curacao. In other words, ensuring that professionals have been appraised and found capable of providing the clinical duties they had been assigned to perform [23]. The focus of the analysis was to clearly describe the educational characteristics of the affiliate teaching hospital and while making use of this environment, achieve a new professional culture i.e. competent and patient centered care, among health care providers of the island.

This was necessary for guaranteeing the quality of both medical education and of health care delivery on the island. The main objectives of this process were first, to identify the different perspectives and organizational requirements required to build a health care environment that was conducive for change and second, to create an environment that would facilitate the smooth migration of existent services into an effective and sustainable value based health care system. The ultimate goal was to have physicians as health care professionals be leaders in the process. Hence, this report demonstrates as a proof of concept, the development and successful introduction of a new health care delivery framework using inputs from the local health care environment and guided by the recommendations for competency based training, derived from the revised Dutch postgraduate medical curriculum [24] Similar to the CanMeds competency framework, seven professional competencies considered essential for practicing physicians were identified and outlined for implementation in the national postgraduate training programs [25].

### Methods

**Figure 1:**
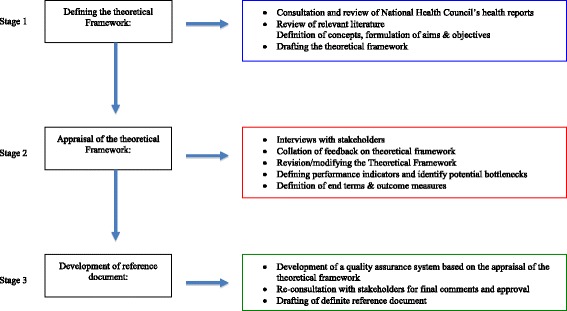
**The stages of the iterative process used in developing the reference document.**

The strategy that we applied in developing and implementing this framework involved the systematic application and synthesis of the existing knowledge of change in health care, the application of expert opinions and the use of methods of qualitative research to obtain information from different stakeholders (triangulation). The approach we used included:

1. Formulating the standards of the quality of health care based on review of national health care council reports, existent literature, consultation with experts and national health care council by a content expert,
2. Formulating a proposal for the reform of the health care environment and services (by selected members of the health care council and the content expert) based on the desired local health care objectives in combination with relevant evidence in the literature. This proposal was designed with a clearly defined aim, framework and process for implementation,
3. Seeking the input, approval and support of important stakeholders (i.e. the national health care council, health care providers e.g. physicians and nurses and funders of care i.e. health care insurers) for the proposal (Figure 1).

#### Formulating the standards for the quality of health care

The project was conducted between the period of September 2010 and April 2011. A content expert drafted a recommendation document defining the requirements for achieving a proper environment for achieving value based medical care in Curaçao. The content expert was a physician manager and educationalist, with a first hand experience of the health care delivery system of the island having trained and practiced in Curaçao. In the first stage of preparation, use was made of relevant reference documents, literature on organizational and health care development as well as the personal professional experience [5,24,26]. Between September and December of 2010, a draft describing the context/environment was developed highlighting the framework for competency-based training for trainee physicians and the continued professional development of practicing health care professionals. The aims and objectives were defined and important stakeholders for consultation during the process were identified.

#### Formulating the reform proposal

A comprehensive pathway for implementing and evaluating the various stages of the proposed recommendations was designed including a timeline for achieving the different steps involved in the process. The second stage of the process involved a qualitative analysis of the proposed standards and pathway aimed at addressing local requirements and testing the feasibility, acceptability and comprehensiveness of the recommendations among the stakeholders and supplementing any omissions found. For this purpose, twelve separate interviews were conducted in December 2010 with the Chief executive officer (CEO) of SEHOS and the CEO’s of 2 small surgical clinics (primarily short stay surgery e.g. ophthalmology and Ear Nose and throat) on the island (all physicians), the clinical directors of 3 specialty departments and the director of the department of epidemiology and biostatics in the St. Elisabeth Hospital, representatives of the two biggest health care insurers, the inspector general of the island’s health care inspectorate, a representative of the association of family physicians and with a delegate of the island’s health care council. The health care council of Curaçao comprised of representatives from the providers, consumers and funder organizations present on the island. Prior to each interview, the stakeholders received a copy of the draft proposal for critical evaluation and requested to provide written feedback on the feasibility, acceptability and comprehensiveness of the content in the proposal. All of the stakeholders provided written feedback on the proposal and also elaborated and clarified their responses in the face-to-face interview that followed. Between January and April 2011, all interviews (60 minutes duration) were conducted and the comments received were transcribed, analyzed and synthesized into the initial proposal (See Table 1). The comments were categorized as relating to a) Professional standards, b) Distribution of responsibilities, c) Accountability, or d) quality assurance and improvement.

#### Seeking approval and support of important stakeholders for the proposal

The last phase of the project involved seeking approval and acceptance of the final drafted proposal. Using the inputs we received from the participating stakeholders, a revised proposal was sent back to each of them for evaluation and approval. This resulted in a more inclusive recommendation that contained inputs from all of the stakeholders and a definite reference document [23].

## Findings

The outcome of our interviews yielded valuable qualitative information that was relevant for the local environment. We summarized these findings into themes, which we subsequently clustered into our general and specific recommendations.

### General recommendations

Our findings revealed that the chosen approach was well accepted by the stakeholders. It also showed that in order to achieve a new value based health care system based on a well-organized and reliable system, the quality of the competence of medical specialists and that of health care system needed to be guaranteed. For this, extra focus would need to be given to improving the quality of

1. Medical practice,
2. (Postgraduate) medical education and training and
3. Continuing professional development of medical specialists

Furthermore, such a system would be required to maintain, promote and safeguard the quality of the educational process and also lead to an integral health care delivery system that offers support to all care delivery and teaching institutions on the island. For this, the system would need to:

1. Safeguard the quality and safety of care in all health care institutions.
2. Define a clear framework for measuring quality using (valid) quality indicators.
3. Identify deficiencies in care and education in an efficient and simple manner, and
4. Create opportunities for innovation in health care and education.

### Specific recommendations

A number of specific recommendations were identified as essential for realizing the above-mentioned general aims. These included the development of and/or efforts to organize:

1. The effective distribution of professional responsibilities
2. Accountability among all stakeholders
3. Professional standards
4. Indicators to assure and improve the quality of care
5. Frameworks to enable specification of educational content and training activities^a^ so that they can be assessed in a practical and efficient manner.
6. Opportunities to develop and demonstrate ‘best practices’.

A more detailed description of the specific recommendations is presented in Table 1.

**Table 1:** **Overview of specific recommendations provided by the different stakeholders**

**1. Professional standards**	**2. Distribution of responsibilities**	**3. Accountability**	**4. Quality assurance and improvement**
Medical specialists and the hospital institutions should both fulfill the requirements expected from good care providers. They should act in accordance with the responsibilities as defined by their respective professional standards.	The medical specialist is to be held primarily accountable for the specialist care he/she provides. He/she is also accountable for the care providers who work under his responsibility.	The medical specialist systematically gives account of the quality of the care he has provided in the manner agreed upon between the executive members of the medical staff and the Board of Directors.	The medical specialists and the hospitals collaborate in assuring quality and improving the care provided in the hospital and also in those activities considered to be in the best interest of the patient, the hospital and/or the medical specialist.
The medical specialist is expected to provide good health care services that meet acceptable standards i.e. are safe, effective, patient centered, delivered timely and commensurate with the patient’s real needs.	The medical specialist shares the responsibility for care with other care providers involved in the health care process. It is possible to set up partnerships/departments and to formulate desired professional responsibilities in regulations or contracts for employment purposes.	The board of the medical staff periodically informs the Supervisory Board about the quality and safety of care via the board of directors (medical director).	The medical specialist participates regularly in formal meetings (such as meetings of the group practice or department) not only within his medical specialty (such as patient hand over, difficult cases, necrology, complications), but also between medical specialties (such as patient handover, necrology, clinical pathology, x-ray, intensive care, trauma and complications).
The medical specialist abides by the professional guidelines/protocols that apply to him, and may deviate from these, if and when necessary, and documenting the reason(s) for this in the medical file. Should a specialist need to deviate from a professional guideline/protocol then the documentation should be in a (predefined) uniform format.	The medical staff and the hospitals board of Directors are jointly responsible for the development of professional standards for medical specialists.	The board of the medical staff and the Board of Directors receive the conclusions and recommendations from internal and external quality assurance audits from partners, department or group practice, and they receive reports of educational audits from the hospital’s central committee for educational activities	In order to ensure the quality of care and education, use must be made of indicators measuring both internal and external parameters.
The medical specialist keeps her knowledge and skills up to date, for instance by attending accredited continuing professional development activities required for re-registration in the register of medical specialists of the Medical Specialist Registration Commission (MSRC) or an equivalent body in Curaçao.	In accordance with the National Legislation on Health Care Institutions, the Board of Directors has the final responsibility for the integral quality of care within the health care institution and must also create the conditions to facilitate the delivery of good care.	The medical specialist takes part in the periodical and systematic evaluations of his personal functioning within the hospital.	• *External Measurement of Quality Assurance* - Characterized by periodic visitations/accreditation. If such a procedure is not established in accordance with an integral “quality of care” system, it often results in expensive and bureaucratic processes with low return and relatively small improvements. In general, such a procedure only promotes compliance with minimal standards aimed at obtaining and maintaining the status of an accredited top clinical institution (for example the NIAZ accreditation) or training hospital (such as CCMS or MSRC recognition) .
The medical specialist keeps a medical file of every patient treated or examined by him in the outpatient clinic, in the hospital ward or in the short-stay clinic in a manner customary to the hospital and keeps a medical file (in a standardized manner ), that provides a good overview of the history of the disease, the diagnosis or diagnoses, the treatment plan and the current situation.	The medical specialist must be aware of the limitations of his/her own capabilities and expertise, and must refer patients timely to other specialists or care providers if and when necessary.	The medical specialist must report incidents in accordance with the procedures of the organization in which he/she works and provides all factual information that is considered to be necessary and in the best interest of the patient and the hospital.	• *Internal Measurement of Quality Assurance* - Characterized by continuous and reliable collection of information that can be used for feedback and improvement of quality. If well designed and organized, such a process is not expensive and yields more results for improvement.
Also, the standardization of medical records/patient files offers benefits for internal and external visitations to monitor the quality of care and the training of medical doctors and medical specialists.	The medical specialist calls to account his colleagues and members of the partnership, department or group practice in cases of undesired behavior and takes steps to rectify any undesirable situation. If necessary, the medical specialist informs the board of the medical staff and/or the Board of Directors.	The medical specialist immediately informs the board of the medical staff and the Board of Directors of any calamities as well as circumstances that could possibly lead to a calamity.	
Reliable registration procedures are also needed from a public health care perspective.	Should there be a suspicion of possible dysfunctional behavior, then this shall be dealt with in accordance with the Dutch document on the professional code regarding potential dysfunctional medical specialists [2] or an equivalent regulation in Curaçao.	Incidents, calamities, complications, errors, near misses and complaints are regularly discussed within departments, group practice or medical staff, and steps for improvement are formulated, implemented and evaluated periodically	
	At all times, it should be clear to the patient:	Openness about medical errors or incidents is a priority within the hospital as well as between medical specialists and in relation to the patient.	
	i. Who they or their guardians can contact;		
	ii. Who is responsible for the care provided to them;		
	iii. Who is responsible for the coordination of (their) care.		
	The medical specialist who together with a colleague or colleagues and/or other care providers provides care to a patient, makes clear arrangements with those colleague(s) and/or care provider(s) about the distribution of tasks and responsibilities concerning the care for the patient. These agreements are documented in writing in the patient’s medical and/or in the nursing file, so that it is clear at all times whom the point of contact is for that patient.	The patient has a right to honest and timely information about the nature and full facts of any incidents that have noticeable consequences for him/her.	
	The medical specialist who, together with other care providers delivers care to a patient, makes sure that he has the relevant data available for those medical specialist(s) or care provider(s) and informs them about any details and findings that they need to be able to provide good care. The medical specialist makes sure that the rights of patients are well protected.	The medical specialist must report about the nature and full facts of incidents that have noticeable consequences for the patient and record this in the patient’s medical file. The medical specialist must immediately report to the board of the medical staff and the Board of Directors if or when civil, criminal or disciplinary actions related to care provided has been taken against him/her.	

## Conclusions

The island of Curaçao is currently at a crucial crossroad within the history of its health care system. Specific choices need to be made regarding the organization and the quality of health care delivery to the inhabitants and visitors of the island. As mentioned earlier in this paper, a proper environment for change is needed to ensure that the quality of medical care on the island meets internationally accepted standards and that the consumers of care can trust the quality of service they receive. Also, a central role for health care reform in the St. Elisabeth Hospital was presented as a key factor in the national process.

While there is an obvious need for ongoing research, development and improvement of the island’s health care systems, it is crucial that all health care providers (HCP) e.g. medical specialists, general practitioners, allied HCP’s such as nurses and care workers are acknowledged as equal partners in the decision making process of the island’s health care system. The same applies to all other parties or organizations that play a role in the health care delivery system, such as boards of health care institutions, insurers and patient and client organizations [27].

In order to arrive at the final recommendations proposed in this project [23], several rounds of consultation with these stakeholders were needed. These consultations focused mainly on how to deal with the complexities of legislation and the local health care system. It also focused on incorporating key points regarding professional standards, distribution of responsibilities, professional accountability, and quality assurance as well as obtaining the approval of stakeholders. The consultations were guided by the draft proposal that was designed using the existing framework for competency-based medical education and continued professional development for practicing health care professionals. A similar approach had already been advocated in formulating realistic local needs assessment for achieving competency based medical education [28]. The needs assessment however, had to reflect the needs of the local community, (in this case that of SEHOS as a teaching and only major hospital serving the community,) and also lead to a sense of ownership among the stakeholders. Both of these processes have been found to have high potential for change in resource-limited settings [28]. Our findings also revealed that the structured consultations based on the framework of a competency based training program effectively facilitated relevant and useful contribution from the local stakeholders.

The recommendations presented in this paper, are in alignment with recently proposed reforms by a global independent commission addressing education of health professionals in the 21st century [4]. However for a successful implementation in practice, there would be the need for an appropriate environment for change and different organizations acting together in unison. Sustenance of the desired health care changes would also demand strong leadership and for this, medical and allied health professionals would need to re-possess a leading role in the health care delivery system (professionals in the lead) [1–3,28,29]. This is because health care professionals are believed to possess and clearly demonstrate the necessary competencies regarding patient-centered care, teamwork and leadership, quality improvement, accountability, cost-consciousness and most of all professionalism [3,28].

As far as we know, this is a first report of the findings from the implementation of a “medical education driven” health care reform in a developing country. The model used to formulate the recommendations in this paper seems to be fit for use in similar small scale communities (with scarce resources) [4] where medical education reform could be key a facilitator in the transformation process. In addition to obtaining a well grounded reference document that would secure the approval and commitment of various actors in the health care education and delivery process i.e. ownership, the incorporation of the various consultation rounds with stakeholders in our model also assured the inclusion of social accountability and the local priority health care needs of the community, with a leading role for medical education in the process [28].

In conclusion, the final document that was produced based on the recommendations in this paper, has been fully integrated into an official development plan [23]. This plan has been approved by the medical staff and hospital management of the SEHOS and is currently being implemented in the hospital. In our view, this is a timely intervention, as a growing area of interest in the literature that requires further investigation, is how teaching institutions can be used to restructure health care [30]. Finally, as shown in this report, we believe that the collaborative (educational) efforts with international medical institutions was of great value for this project. Particularly, in preparing and strengthening an educational infrastructure capable of addressing the needs of the resource limited environment described in this paper.

## Endnote

^a^This refers to both post- and undergraduate medical education of residents and students and to continuing professional development of specialists.

## Abbreviations

UMCG: University Medical Center Groningen
SEHOS: St. Elisabeth Hospital
NAF: Netherlands Antilles Florijn
USD: United States Dollar
GDP: Gross domestic product
CEO: Chief Executive Officer
HCP: Health Care Provider(s)
WHO: World Health Organization
